# Cannabinerol Prevents Endoplasmic Reticulum and Mitochondria Dysfunctions in an In Vitro Model of Alzheimer’s Disease: A Network-Based Transcriptomic Analysis

**DOI:** 10.3390/cells13121012

**Published:** 2024-06-10

**Authors:** Luigi Chiricosta, Aurelio Minuti, Agnese Gugliandolo, Stefano Salamone, Federica Pollastro, Emanuela Mazzon, Osvaldo Artimagnella

**Affiliations:** 1 IRCCS Centro Neurolesi “Bonino-Pulejo”, Via Provinciale Palermo, Contrada Casazza, 98124 Messina, Italy; 2 Department of Pharmaceutical Sciences, University of Eastern Piedmont, Largo Donegani 2, 28100 Novara, Italy; salamone.ste@gmail.com (S.S.); federica.pollastro@uniupo.it (F.P.)

**Keywords:** Alzheimer’s disease, cannabinerol, phytocannabinoids, transcriptomic analysis, network analysis, mitochondria, endoplasmic reticulum

## Abstract

Neurodegenerative disorders are affecting millions of people worldwide, impacting the healthcare system of our society. Among them, Alzheimer’s disease (AD) is the most common form of dementia, characterized by severe cognitive impairments. Neuropathological hallmarks of AD are β-amyloid (Aβ) plaques and neurofibrillary tangles, as well as endoplasmic reticulum and mitochondria dysfunctions, which finally lead to apoptosis and neuronal loss. Since, to date, there is no definitive cure, new therapeutic and prevention strategies are of crucial importance. In this scenario, cannabinoids are deeply investigated as promising neuroprotective compounds for AD. In this study, we evaluated the potential neuroprotective role of cannabinerol (CBNR) in an in vitro cellular model of AD via next-generation sequencing. We observed that CBNR pretreatment counteracts the Aβ-induced loss of cell viability of differentiated SH-SY5Y cells. Moreover, a network-based transcriptomic analysis revealed that CBNR restores normal mitochondrial and endoplasmic reticulum functions in the AD model. Specifically, the most important genes regulated by CBNR are related mainly to oxidative phosphorylation (*COX6B1*, *OXA1L*, *MT-CO2*, *MT-CO3*), protein folding (*HSPA5*) and degradation (*CUL3*, *FBXW7*, *UBE2D1*), and glucose (*G6PC3*) and lipid (*HSD17B7*, *ERG28*, *SCD*) metabolism. Therefore, these results suggest that CBNR could be a new neuroprotective agent helpful in the prevention of AD dysfunctions.

## 1. Introduction

According to the World Health Organization (WHO), across the world, 55 million people suffer from Alzheimer’s and other dementias, with the numbers expected to increase to 139 million in 2050 due to population aging [1]. The global impact of dementia affects not just patients but also caregivers and healthcare systems.

Alzheimer’s disease (AD) is a progressive neurodegenerative disorder and the most common form of dementia. It is characterized by severe cognitive decline, including memory, language, and behavioral impairments, and is often accompanied by motor disabilities [2,3]. Molecularly, it is characterized by the accumulation of extracellular deposits of β-amyloid (Aβ), intracellular tau-containing neurofibrillary tangles, microglia activation, and mitochondrial and endoplasmic reticulum dysfunctions. Altogether, this leads to the accumulation of misfolded/unfolded proteins, alterations in calcium homeostasis, oxidative stress, and apoptosis [2,4]. Studies on AD patients and AD models reported a reduction in mitochondrial activity and ATP production, even before plaque formation [5]; moreover, it has been shown that the accumulation of Aβ-peptides leads to prolonged endoplasmic reticulum stress linked with the dysregulation of mitochondrial metabolism [6,7], contributing to the onset and development of AD [8,9].

To date, there are no definitive therapies against AD, but available medications help to reduce the symptoms and clinical decline. Two types of drugs are recommended by the U.S. Food and Drug Administration (FDA) for the treatment of AD: pharmacological drugs and monoclonal antibodies. The former are mainly used to alleviate AD symptoms; they include donepezil, galantamine, and rivastigmine, which inhibit acetylcholinesterase activity, and memantine, an antagonist of the N-methyl-D-aspartate (NMDA) receptor [10]. In contrast, the latter include aducanamab, lecanemab, and donanemab (in the FDA approval phase), which are able to bind to and reduce the levels of Aβ at different stages of plaque formation [11]. Other drugs are under consideration, including γ- and β-secretase inhibitors, α-secretase modulators, and tau protein aggregation inhibitors (full list and details reviewed in [12]). In addition to medical therapies, prevention strategies, such as lifestyle improvement and neuroprotective molecules, are helpful in reducing risk factors and protecting neurons from degeneration.

In this regard, cannabinoids have recently been shown to have neuroprotective properties in diverse neurodegenerative diseases, including AD [13,14,15,16]. Specifically, Δ^9^-tetrahydrocannabinol (Δ^9^-THC), the most abundant phytocannabinoid of Cannabis sativa, has been demonstrated to inhibit acetylcholinesterase activity [17], and it reduces glutamate-mediated hyperexcitability in primary neuronal cultures [18]. Moreover, it increased neprilysin levels, the endopeptidase responsible for Aβ degradation, reducing Aβ accumulation in 5xFAD transgenic mice [19]. However, Δ^9^-THC also displayed psychotic effects [20], limiting its clinical applicability. In addition to Δ^9^-THC, other phytocannabinoids have been investigated in AD models. Cannabidiol (CBD), a non-psychoactive compound, was reported to protect rat PC12 neurons from Aβ-induced toxicity [21] and to inhibit tau hyperphosphorylation [22]. In vivo studies showed that long-term oral treatment with CBD prevented memory impairments in the APP × PS1 transgenic mouse AD model [23] and delayed cognitive decline in 5xFAD transgenic mice [24]. In an in vitro AD model, cannabigerol (CBG) prevented the accumulation of the Aβ protein; in addition, cannabidivarin (CBDV) reduced Aβ toxicity and prevented energy loss [25]. Finally, cannabidiolic acid (CBDA) and tetrahydrocannabinolic acid (THCA) have also been shown to reduce Aβ and tau pathologies, improving memory in a murine AD model [26].

Recently, “minor cannabinoids” [27], such as ∆^8^-tetrahydrocannabinol (Δ^8^-THC) and cannabinerol (CBNR), have also been investigated to understand whether they have similar neuroprotective functions compared to “major ones”. Accordingly, our group has previously documented that Δ^8^-THC pretreatment of differentiated SH-SY5Y neurons prevented the Aβ-induced loss of cell viability by modulating genes involved in endoplasmic reticulum stress and proteostasis, thus reducing apoptosis [28]. Moreover, Δ^8^-THC was also reported to moderately inhibit acetylcholinesterase and butyrylcholinesterase activity [29]. CBNR, a geometric isomer of CBG, is a less-studied phytocannabinoid that we recently demonstrated to be involved in neuronal differentiation in NSC-34 cells [30] and in the regulation of genes associated with synapse organization and specialization, such as cytoskeleton- and ion channel-related genes [31].

In this study, we evaluated the potential neuroprotective role of CBNR and the underlying molecular mechanisms in an in vitro cellular model of AD via next-generation sequencing. A network-based transcriptomic analysis was performed to understand which subcellular structures and genes could be modulated by CBNR in the neuroprotection and prevention against AD dysfunctions.

## 2. Materials and Methods

### 2.1. Cell Culture and Differentiation

The human neuroblastoma cell line SH-SY5Y was obtained from the American Type Culture Collection (ATCC) (Manassas, VA, USA). Cells were plated onto 96-well plates (#353072, Corning Incorporated, Corning, NY, USA) at a density of 50,000/well or onto 6-well plates (#130184, Thermo Scientific, Rochester, NY, USA) at a density of 800,000/well in maintenance medium composed of DMEM/F-12 Ham (#D6421, Sigma-Aldrich, Saint Louis, MO, USA) supplemented with 10% fetal bovine serum (FBS) (#F7524, Sigma-Aldrich, Saint Louis, MO, USA), 1% penicillin/streptomycin (#P0781, Sigma-Aldrich, Saint Louis, MO, USA), and 1% L-glutamine (#G7513, Sigma-Aldrich, Saint Louis, MO, USA) at 37 °C in a humidified atmosphere of 5% CO2 and 95% air. On the following day, SH-SY5Y cells were incubated for 5 days with 10 µM of retinoic acid (RA) (#R2625, Sigma-Aldrich, Saint Louis, MO, USA) in order to induce differentiation. RA-containing medium was refreshed midway through the treatment.

### 2.2. Cell Characterization via Western Blot Analysis

At the end of the RA treatment, SH-SY5Y differentiated cells and sister SH-SY5Y non-differentiated cells were harvested with ethylenediaminetetraacetic acid (EDTA) for 5 min at 37 °C. Then, the cells were pelleted and proteins were extracted using RIPA buffer (#89901, Thermo Scientific, Waltham, MA, USA) according to the manufacturer’s instructions. Western blot analysis was performed according to standard methods. The protein concentration was evaluated using the Bio-Rad Protein Assay (#5000006, Bio-Rad Laboratories, Hercules, CA, USA), and thirty micrograms of proteins were denaturated in β-Mercaptoethanol-added Sample Buffer 1X (#161-0747, Bio-Rad Laboratories, Hercules, CA, USA) at 95 °C for 5 min. Next, proteins were separated on sodium dodecyl sulfate–polyacrylamide gel electrophoresis (SDS-PAGE) and transferred onto a PVDF transfer membrane of 0.45 µm (#IPVH00010, Immobilon-P PVDF, Merck Millipore division of Merck KGaA, Darmstadt, Germany). The membrane was blocked for 1 h at room temperature in 1X TBS-Tween containing 5% non-fat dried milk. Then, it was incubated with the primary antibody overnight at 4 °C. Afterwards, the membrane was washed 3 times in 1X TBS-Tween, incubated 1 h with horseradish peroxidase (HRP)-conjugated secondary antibody in 1X TBS-Tween containing 5% non-fat dry milk at room temperature, washed again 3 times, and the signal was finally revealed by an enhanced chemiluminescence reagent (#WBLUF0500, Immobilion Forte Western HRP Substrate, Millipore Corporation, Burlington, MA, USA). The following primary antibodies were used: anti-AChRα1, rat monoclonal (1:200; #sc-65829, Santa Cruz Biotechnology, Inc., Dallas, TX, USA); anti-TH, rabbit polyclonal (1:1000; #AB152, Millipore, Burlington, MA, USA); anti-Neurofilament, mouse monoclonal, clone DA2 (1:1000; #MAB1615, Millipore, Burlington, MA, USA); in NSC-34 cells and HRP-conjugated anti-GAPDH, rabbit monoclonal, 14C10 (1:1000; #3683, Cell Signaling Technology, Danvers, MA, USA).

The following secondary antibodies were used: HRP-conjugated mouse anti-rabbit IgG (1:2000; #sc-2357, Santa Cruz Biotechnology, Inc., Dallas, TX, USA), HRP-conjugated chicken anti-mouse IgG (1:2000; #SA1-72021, Thermo Scientific, Waltham, MA, USA), and HRP-conjugated goat anti-rat IgG (1:2000; #a110-105p, Bethyl Laboratories, Montgomery, TX, USA). The same membrane was processed for each pair of primary and secondary antibodies, then stripped with Restore^TM^ Western Blot buffer (#21059, Thermo Scientific, Meridian, Rockford, IL, USA).

Images were acquired with a ChemiDoc™ MP System (Bio-Rad Laboratories S.r.l., Hercules, CA, USA) and analyzed with the ImageJ-Fiji 1.54f software. Protein levels were normalized against GAPDH. The uncropped blots for TH, Neurofilament, AChRα1, and GAPDH are available in Supplementary Figures S1, S2, S3, and S4, respectively.

### 2.3. Cell Treatment with β-amyloid and CBNR

After 5 days of RA-induced differentiation, SH-SY5Y cells were pretreated for 24 h with CBNR at 10 µM or 20 µM (two concentrations that have been previously reported to show biological effects and no cytotoxicity [30,31]) in maintenance medium. CBNR was provided by the Department of Pharmaceutical Sciences, University of Eastern Piedmont, dissolved in dimethylsulfoxide (DMSO) (#D8418, Sigma-Aldrich, Saint Louis, MO, USA), and further diluted in phosphate-buffered saline (PBS) 1× (#806552, Sigma-Aldrich, Saint Louis, MO, USA). β-amyloid peptide 1-42 (Aβ) (#A9810, Sigma-Aldrich, Saint Louis, MO, USA) was also dissolved in DMSO, diluted in PBS 1×, and finally aggregated at 37 °C for 24 h. It has been demonstrated that Aβ incubation for 24 h at 37 °C induces the formation of aggregates [32]. At the end of the pretreatment, cells were treated with 10 µM of Aβ for 24 h. Control cells were simply treated with PBS-diluted DMSO in the maintenance medium. The final DMSO concentration in cell cultures was <0.1%.

### 2.4. MTT Assay

SH-SY5Y cell cultures were set up and treated in 96-well plates as described in “Cell Culture and Differentiation” (Section 2.1) and “Cell Treatment with β-amyloid and CBNR” (Section 2.3). At the end of the treatment, cell metabolic activity was evaluated with a Thiazolyl Blue Tetrazolium Bromide (MTT) assay (#M5655, Sigma-Aldrich, Saint Louis, MO, USA). Briefly, cells were incubated with the maintenance medium containing 0.5 mg/mL MTT at 37 °C for 4 h. The formed formazan crystals were dissolved in acidic (0.1 N HCl) isopropanol at 37 °C for 1 h to produce a colored solution. The absorbance was quantified by spectrophotometric measurement at 570 nm using the BioTek Synergy H1 microplate reader (Agilent, Santa Clara, CA, USA). The background was measured at 630 nm.

### 2.5. Extraction of Total RNA and cDNA Library Preparation

SH-SY5Y cell cultures were set up and treated in 6-well plates as described in “Cell Culture and Differentiation” (Section 2.1) and “Cell Treatment with β-amyloid and CBNR” (Section 2.3). At the end of the treatment, cells were harvested using 0.25% trypsin-ethylenediaminetetraacetic acid (EDTA) solution (#T4049, Sigma-Aldrich, Saint Louis, MO, USA) and pelleted by centrifugation (300× *g* for 5 min). Then, the pellet was processed for RNA extraction. Total RNA was extracted using the Maxwell^®^ RSC simplyRNA Cells Kit (#AS1390, Promega, Madison, WI, USA) with the Maxwell^®^ RSC instrument. Afterwards, library preparation (from 100 ng of total RNA) of two biological replicates was carried out using TruSeq^®^ RNA Exome (#20020492, #20020189, #20020490, #20020183, Illumina, San Diego, CA, USA) [31], following the manufacturer’s instructions as already reported. The Tapestation 4150 instrument (Agilent, Santa Clara, CA, USA), using the D1000 screentape (#5067-5582 and #5067-5583, Agilent, Santa Clara, CA, USA), was used to validate the quality of the library. A denaturation step using 0.2 N sodium hydroxide (NaOH) was performed, and then it was diluted until it reached a concentration of 1.42 pM. The NextSeq 500/550 Mid Output Reagent Kit v2 (300 cycles) (#20024905, Illumina, San Diego, CA, USA) was used for sequencing with the Illumina instrument NextSeqTM 550Dx (Illumina, San Diego, CA, USA). The run was made in the paired-end mode.

### 2.6. Comparative Transcriptomic Analysis

The quality of the sequence data acquired from the sequencer was tested with the FastQC tool version 0.11.9 (Babraham Institute, Cambridge, UK). Subsequently, Trimmomatic version 0.40-rc1 [33] (Usadel Lab, Aachen, Germany) was exploited for the removal of adapters and low-quality bases. The refined reads were used as input for the STAR RNA-seq aligner 2.7.3a [34] (New York, NY, USA) against the human reference genome (GRCh38). Following alignment, the expression level of the various transcripts was determined utilizing the HTSeq-count Python package version 0.13.5 [35] (European Molecular Biology Laboratory (EMBL) in Heidelberg, Germany). Differentially expressed genes (DEGs) were identified through the DESeq2 library version 1.36.0 [36] in R version 4.2.0 (R Core Team). Corrected *p*-values, defined as *q*-values, were computed using the Benjamini–Hochberg post procedure, and a threshold of 0.05 was set to minimize the number of false positive genes. Data manipulation and plots were generated using the R libraries dplyr (version 1.1.4), ggplot2 (version 3.4.0), and circlize (version 0.4.15).

### 2.7. Gene Network Analysis

We used Panther ([37], available online: https://pantherdb.org, accessed on 7 February 2024) to retrieve cellular component terms from the gene ontology. We performed an overrepresentation analysis of our DEGs against the *Homo sapiens* reference list using default parameters (Fisher’s exact test corrected by the false discovery rate). There were 45 overrepresented terms, among which one is related to an unclassified ontology (Table S3). Thus, we inspected the deepest child of each positive overrepresented cluster. For each of the 11 terms, we built the network using the STRING database ([38], available online: https://string-db.org/, accessed on 7 February 2024). The limit of nodes in a STRING network is 2000; thus, we excluded larger networks. We used the most pressing interaction score (0.900). All other parameters chosen are the default ones. For each network, we downloaded from STRING the tabular text output (string_interactions.tsv), and we performed the analysis of the network using R (version 4.2.0). Specifically, the library “igraph” (version 1.4.1) was used for constructing the topology of the network and for computing the betweenness centrality (*BC*), the closeness centrality (*CCe*), the neighborhood connectivity (*NC*), the clustering coefficient (*CCo*), the average shortest path (*AVP*), and the number of vertices (*V*) for each node of the network. For each module of each network, the weight (*W*) associated to each DEG was computed using the following formula:

$$W=\frac{BC\times CCe\times \log_{2}NC\times CCo}{2^{-\log_{2}V}\times AVP}$$

Some particular cases were observed. In particular, if a gene had either a *BC*, *CCe*, or *CCo* equal to 0, we substituted 0 with the lowest value of the *BC*, *CCe* or *CCo* (depending on which of them is 0) in the network in which it took place. This substitution is necessary in order to prevent a biased null weight. In the case in which all the values of the parameter were 0 for a network, the 10^−7^ value was set. Indeed, this value is lower than the lowest value.

### 2.8. Statistical Analysis

**Figure 1:**
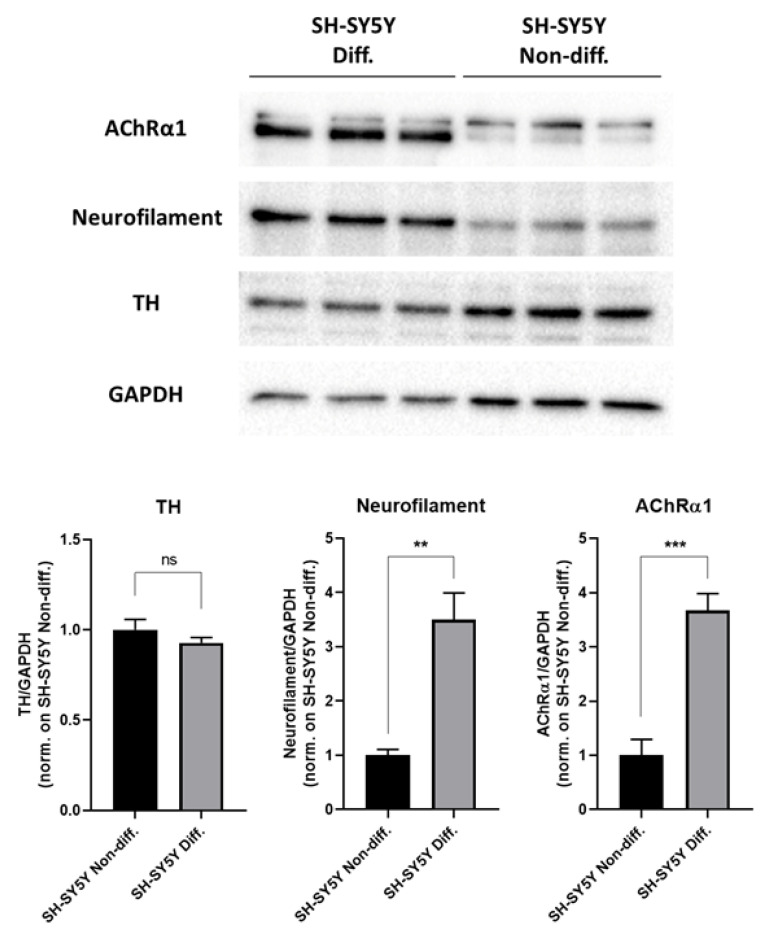
Characterization of RA-differentiated SH-SY5Y cells. Differentiated and non-differentiated SH-SY5Y cells were compared, and the protein levels of TH, Neurofilament, and AChRα1 neuronal markers were evaluated via Western blot assays. Protein levels are doubly normalized against GAPDH and SH-SY5Y non-differentiative cells and expressed as the mean ± SD. There were three biological replicates per condition. ** *p* < 0.01; *** *p* < 0.001; *ns* stands for not significant. The complete primary data and statistical analysis are reported in Table S1.

**Figure 2:**
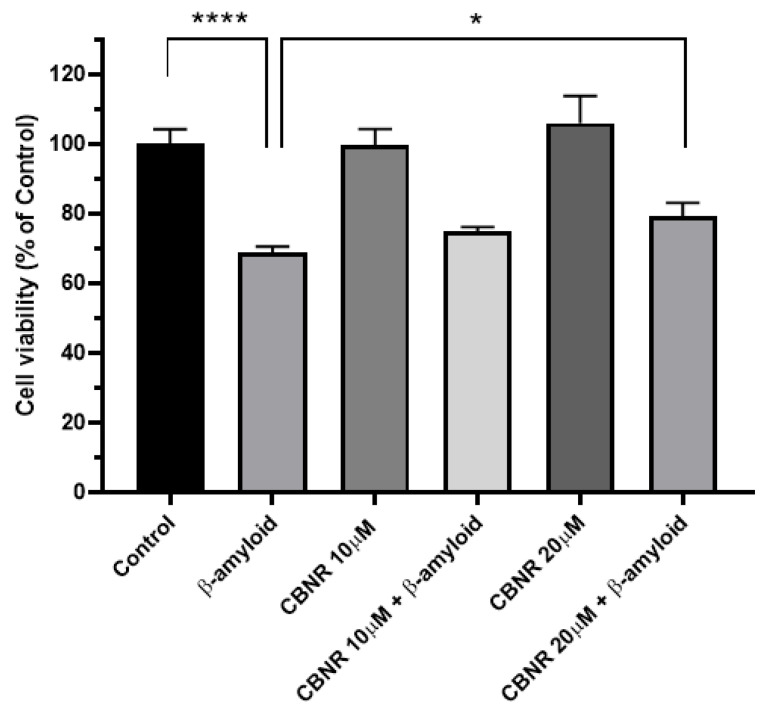
Evaluation of cell viability via the MTT assay after CBNR pretreatment in a cellular AD model. The treatment with Aβ reduced the cell viability of RA-differentiated SH-SY5Y cells, but 20 µM CBNR pretreatment was able to restore it. Results are normalized against the control and expressed as the mean ± SD. There were five biological replicates per condition. * *p* < 0.05; **** *p* < 0.0001. The complete primary data and statistical analysis are reported in Table S1.

The statistical analysis of the cell metabolic activity assay was carried out using GraphPad Prism version 8.0.2 software (GraphPad Software, La Jolla, CA, USA). Multiple comparisons were performed using a one-way ANOVA test and the Bonferroni post hoc test. A *p*-value of less than 0.05 was considered statistically significant. The results are expressed as the mean ± standard deviation (SD). Details of the normalization criteria and number of biological replicates are provided in the legends of Figure 1 and Figure 2. Full primary data are provided in Table S1.

## 3. Results

### 3.1. SH-SY5Y Cell Differentiation and Characterization

As widely reported in literature, retinoic acid (RA) differentiation of SH-SY5Y cells leads to mainly cholinergic neurons, justifying their use as a cellular model of Alzheimer’s disease (AD) [39,40]. We further validated this model by characterizing RA-differentiated SH-SY5Y cells via Western blot analysis. Specifically, as depicted in Figure 1, we compared protein levels of Tyrosine hydroxylase (TH), Neurofilament, and the Nicotinic acetylcholine receptor subunit alpha 1 (AChRα1) between differentiated and non-differentiated SH-SY5Y cells. Results showed that the Neurofilament and AChRα1 proteins, but not the TH protein, were highly upregulated in differentiated cells compared with non-differentiated ones, confirming that RA-differentiated SH-SY5Y cells are mature cholinergic neurons.

### 3.2. CBNR Improves the Aβ-Induced Loss of Cell Viability

In order to evaluate the effects of CBNR in a cellular model of AD, we performed an MTT assay of RA-differentiated SH-SY5Y cells treated with β-amyloid (Aβ) and 10 µM or 20 µM CBNR. As illustrated in Figure 2, Aβ treatment reduced (−31.2%; *p* < 0.0001) the cell viability compared with the control. This Aβ reduction is partially restored (+10.5%; *p* < 0.0142) by pretreating cells with 20 µM CBNR with respect to the Aβ condition. Moreover, CBNR alone is not cytotoxic at both concentrations.

To examine the molecular mechanisms underlying the neuroprotective function of CBNR in this AD model, we analyzed the transcriptomic profile of RA-differentiated SH-SY5Y cells treated with Aβ and pretreated with 20 µM CBNR (CBNR20-Aβ sample), treated with Aβ alone (Aβ sample), or a control (CTRL sample).

### 3.3. Subcellular Structures Regulated by CBNR in the Aβ-Induced AD Model

We performed comparative analyses of the Aβ sample against the CBNR20-Aβ sample (Aβ vs. CBNR20-Aβ) and of the CTRL sample against the Aβ sample (CTRL vs. Aβ). Aβ vs. CBNR20-Aβ showed 1549 differentially expressed genes (DEGs), among which 822 were upregulated and 727 were downregulated. On the other hand, CTRL vs. Aβ resulted in 1000 upregulated and 1262 downregulated genes for a total of 2262 DEGs. All DEGs for both comparative analyses are included in Table S2.

**Figure 3:**
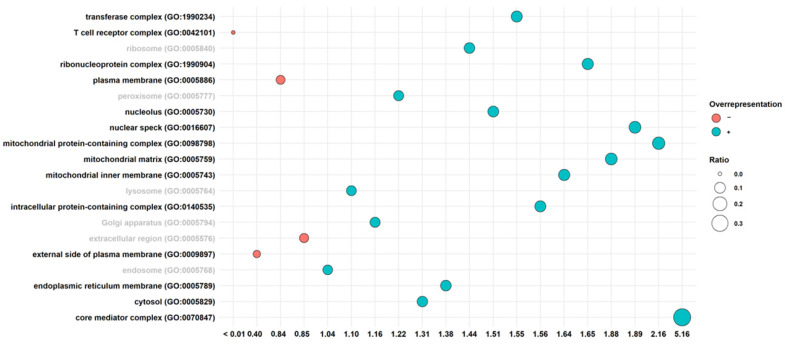
Overrepresentation analysis of cellular component terms in the Aβ vs. CBNR20-Aβ comparison. The score value on the *x*-axis stands for fold enrichment. The black label refers to ontology terms that were statistically significant. Conversely, the gray labels show the terms without any statistical significance. The color of the balls indicates a positive (blue) or a negative (orange) overrepresentation. The size of the balls is related to the ratio between the DEGs found in the ontology terms and the total genes included in the term itself. The terms are sorted alphabetically.

In order to investigate in which subcellular structures CBNR is able to carry out its effects, we performed the statistical overrepresentation of gene ontology terms of the cellular component category for the Aβ vs. CBNR20-Aβ comparative analysis (Table S3). The inspection resulted in 45 overrepresented terms. Among them, we observed the most specific children for each term. In detail, the overrepresented terms were “core mediator complex (GO:0070847)”, “mitochondrial protein-containing complex (GO:0098798)”, “nuclear speck (GO:0016607)”, “mitochondrial matrix (GO:0005759)”, “mitochondrial protein-containing complex (GO:0098798)”, “ribonucleoprotein complex (GO:1990904)”, “mitochondrial inner membrane (GO:0005743)”, “intracellular protein-containing complex (GO:0140535)”, “transferase complex (GO:1990234)”, “nucleolus (GO:0005730)”, “endoplasmic reticulum membrane (GO:0005789)”, “cytosol (GO:0005829)”, “external side of plasma membrane (GO:0009897)”, and “T cell receptor complex (GO:0042101)”. On the other hand, to know where the activity of CBNR was not prominent, we also retrieved the cellular component terms that were not overrepresented to look at the significance of the other subcellular structures. In particular, we focused our attention on “ribosome (GO:0005840) “, “peroxisome (GO:0005777)”, “lysosome (GO:0005764)”, “Golgi apparatus (GO:0005794)”, “extracellular region (GO:0005576)”, and “endosome (GO:0005768)”. Additionally, in Figure 3, we represented all these terms along their scores in the analysis of the overrepresentation.

**Figure 4:**
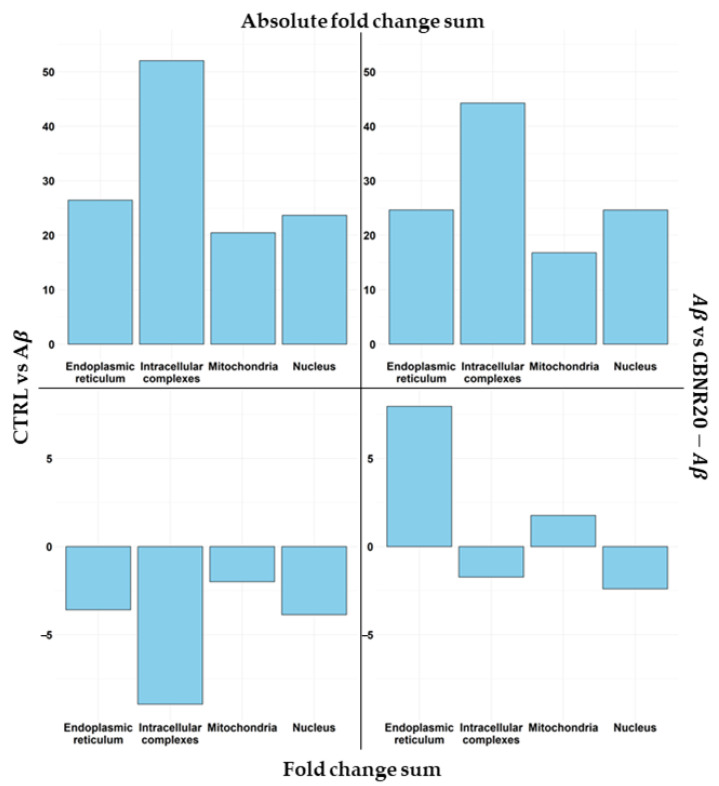
Comparison of DEG regulation among the main subcellular structures. Each plot shows the sum of the fold change for each DEG included in the structure for the CTRL vs. Aβ (left frames) against the Aβ vs. CBNR20-Aβ (right frames) analyses. In the top frames, the sum of the absolute fold change is highlighted, so it is possible to observe how much CBNR is able to regulate the structures. On the other hand, the bottom frames highlight the sum of the positive or negative fold change, showing if the structure is upregulated or downregulated overall.

Successively, we analyzed the impact of CBNR on the diverse subcellular structures as well as which DEGs play a pivotal role in the structure itself. For this purpose, we used a combinatory approach that took into consideration both comparative transcriptomic analyses and a network inspection. First of all, we focused our attention on the overrepresented terms. Among them, we also excluded the terms GO:0009897, GO:0005886, and GO:0042101, which, even with statistical significance, had a negative overrepresentation. Moreover, we had to exclude the term GO:0005829 since it is composed of more than 2000 genes, and it is not feasible to build the network. Then, we categorized the remaining terms in the nucleus (GO:0005730, GO:0016607, GO:0070847), mitochondria (GO:0005743, GO:0005759, GO:0098798), endoplasmic reticulum (GO:0005730, GO:0016607, GO:0070847), and intracellular complexes (GO:0140535, GO:1990904, GO:1990234). In Figure 4, we reported how CBNR is able to regulate the different structures based on the level of the fold change in our AD model. In particular, in order to compare the regulation exerted by CBNR pretreatment with respect to the Aβ condition, we performed a comparison of the CTRL vs. Aβ (left frames) against the Aβ vs. CBNR20-Aβ (right frames) analyses with regard to the DEGs included in the structures regulated by CBNR. In detail, the top frames of Figure 4 show the sum of the absolute fold change, highlighting how much each structure is upregulated or downregulated. Conversely, the bottom frames describe how each structure is modulated. Of note, the distribution of the absolute fold changes in the comparison (CTRL vs. Aβ against Aβ vs. CBNR20-Aβ) is similar among the structures (top frames). Nevertheless, the sign of the regulation is quite different, taking into consideration the actual fold change (bottom frames). These results suggest that the treatment with CBNR in an Aβ-induced AD model counteracts the downregulation observed in the main subcellular structures in Aβ samples, especially the endoplasmic reticulum.

**Figure 5:**
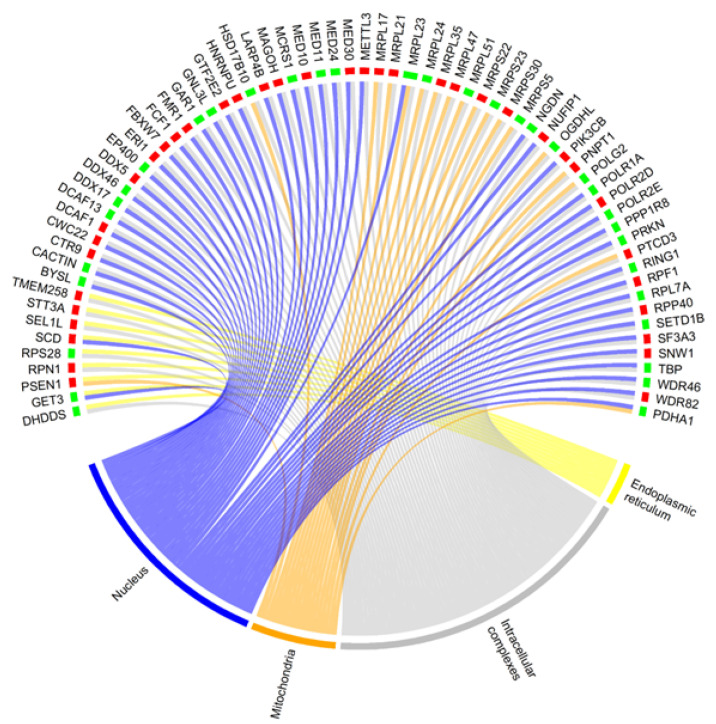
Chord plot representing the DEGs shared among subcellular structures of the Aβ vs. CBNR20-Aβ comparison. Whether a gene is downregulated (green) or upregulated (red) is highlighted at the bottom of each DEG. The structures are the endoplasmic reticulum (yellow), mitochondria (orange), nucleus (purple,) and intracellular complexes (grey).

It is interesting to note that some proteins may overlap their functionality among the subcellular structures of a cell. Indeed, as expected, considering the DEGs of the Aβ vs. CBNR20-Aβ comparison, we observed that around one-third of the DEGs of intracellular complexes were shared with the other structures. Among the shared DEGs, more than one-half are included both in the intracellular complexes and in the nucleus (Figure 5, Table S4).

### 3.4. Network-Based Analysis Reveals Key Genes Regulated by CBNR in the Aβ-Induced AD Model

Every protein has a different relevance in the cellular compartment in which it plays a role. Accordingly, we inspected the interaction network of proteins in each subcellular structure. For this reason, we performed a network analysis, taking advantage of protein–protein interaction networks available in the STRING database ([38], available online: https://string-db.org/, accessed on 7 February 2024).

**Figure 6:**
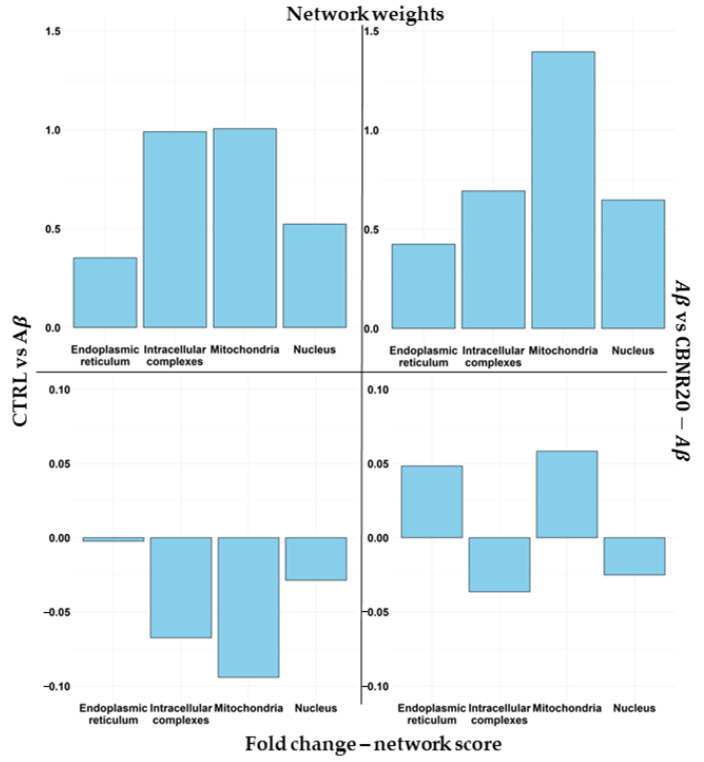
Comparison of weights and weighted-DEG regulation among the main subcellular structures. Each plot includes information about the score for each gene included in the structure for the CTRL vs. Aβ (left frames) and Aβ vs. CBNR20-Aβ (right frames) analyses. In the top frames, the mean of the simple weights is highlighted. On the other hand, the bottom frames show the mean of the naïve fold change combined with the score weight obtained in the network analysis.

In detail, for each cellular component term, we built the protein network using the tightened parameters to limit, as much as possible, the number of weak interactions reported in STRING. Thus, for each network, we computed the parameters “betweenness centrality”, “closeness centrality”, “neighborhood connectivity”, “clustering coefficient”, “average shortest path”, and “number of vertices”. All these parameters (collected in Table S4) were used to assign a score weight of importance to each DEG in our comparative analyses (see the “Materials and Methods” section for further details). Again, we categorized ontologies as mitochondria, endoplasmic reticulum, nucleus, and intracellular complexes, considering the highest weight for those genes shared among more ontologies pertaining to the same category. Next, we observed in Figure 6 (top frames) how the weights of DEGs are distributed among the subcellular structures (top frames), along with the combination of the network score weight with the fold changes (bottom frames). Interestingly, the mitochondria contained DEGs with a higher score and, consequently, were very important in the network. Remarkably, the fold change combined with the score obtained by the network analysis showed that mitochondrial DEGs were highly inhibited in the CTRL vs. Aβ comparison, whereas in Aβ vs. CBNR20-Aβ, the final regulation in the mitochondria looked comparable to that in the endoplasmic reticulum.

In Table 1, we listed the top 10% DEGs for each structure resulting from the network analysis of the Aβ vs. CBNR20-Aβ comparison. In particular, genes involved in oxidative phosphorylation (*COX6B1*, *OXA1L*, *MT-CO2*, *MT-CO3*), protein folding (*HSPA5*), glucose (*G6PC3*) and lipid metabolism (*HSD17B7*, *ERG28*, *SCD*), and the ubiquitin complex (*CUL3*, *FBXW7*, *UBE2D1*) were upregulated. These results suggest that CBNR improves the Aβ-induced dysfunctions with regard to the metabolic activity of mitochondrial and endoplasmic reticulum structures.

## 4. Discussion

Alzheimer’s disease (AD) is a progressively fatal neurodegenerative disorder affecting millions of people worldwide and is still without a definitive treatment. To date, pharmacological drugs mainly act on AD symptoms, whereas FDA-approved monoclonal antibodies try to reduce Aβ accumulation and the clinical decline [10,11]. In this scenario, prevention strategies are helpful to protect neurons from degeneration. Accordingly, many cannabinoids have been studied for their neuroprotective properties in both in vitro and in vivo AD models [13,14,15,16]. They are able to reduce Aβ accumulation and tau hyperphosphorylation, inhibit acetylcholinesterase activity, reduce excitotoxicity, and some mitochondrial and endoplasmic reticulum dysfunctions. Interestingly, we have previously reported that the Δ^8^-THC pretreatment of differentiated SH-SY5Y neurons restored the Aβ-induced loss of cell viability, modulating genes involved in endoplasmic reticulum stress and proteostasis [28]. Δ^8^-THC belongs to the so-called “minor phytocannabinoids”, including the less-investigated CBNR. Recently, we demonstrated in NSC-34 cells that CBNR is involved in neuronal differentiation [30], regulating synaptic genes related mainly to the cytoskeleton and ion channels [31]. Given the role exerted by the other cannabinoids against AD dysfunctions, we also wondered about CBNR.

In this study, we evaluated whether and how CBNR displays a neuroprotective role in an in vitro cellular model of AD. For this purpose, we used a retinoic acid (RA)-differentiated human neuroblastoma SH-SY5Y cell line pretreated with CBNR for 24 h, and then the AD model was induced via the administration of Aβ for an additional 24 h. SH-SY5Y cells are widely used as a cellular model of AD [39,40] due to their ability to differentiate mainly into cholinergic neurons under RA treatment. We further characterized RA-differentiated SH-SY5Y cells, showing high expression of the Neurofilament and AChRα1 proteins, but not of TH, in differentiated cells compared with non-differentiated ones (Figure 1, Table S1). As expected, RA-differentiated SH-SY5Y cells are mature cholinergic neurons.

We observed that pretreatment with CBNR at 20 µM was able to counteract the Aβ-induced reduction of cell viability (Figure 2, Table S1), suggesting an effective neuroprotective role of CBNR at this concentration. In order to molecularly understand how CBNR improves the Aβ damage, we used a next-generation sequencing approach, comparing and analyzing the transcriptome of Aβ samples pretreated with 20 µM CBNR (CBNR20-Aβ) compared with Aβ pretreatment alone. We found 1549 differentially expressed genes (DEGs) (Table S2). Firstly, we focused our attention on subcellular compartments and structures that were mainly regulated by CBNR. The GO analysis (Figure 3) revealed that many of these DEGs belong to the nucleus, mitochondria, and endoplasmic reticulum organelles and to intracellular complexes, such as ribonucleoprotein and transferase complexes (Table S3). Moreover, considering the fold change, CBNR positively regulated the most of the DEGs included in the endoplasmic reticulum as well as the mitochondria. On the contrary, CBNR seemed to negatively regulate the majority of DEGs included in the nucleus and intracellular complexes (Figure 4, bottom right). To compare the effects of CBNR with the Aβ samples, we also considered DEGs (a total of 2262; Table S2) that resulted from the sample control (CTRL) against Aβ and that were included in the structures regulated by CBNR. Interestingly, Aβ negatively regulated all nucleus, mitochondria, endoplasmic reticulum organelles and intracellular complexes (Figure 4, bottom left). These results suggest that treatment with CBNR in an AD model counteracts the global downregulation of the main cellular structures, especially the endoplasmic reticulum and mitochondria.

However, there are two important considerations to take into account in our study: (1) proteins may play their role (directly or indirectly) in more subcellular structures (as we noted for our DEGs in Figure 5); (2) each protein has a different impact on the cell physiology, even according to interactors in the structure to which they belong. In order to address these two points and to investigate the main genes regulated by CBNR to prevent Aβ-induced dysfunctions, we took advantage of the protein–protein interaction networks available on the STRING database [38]. We evaluated the impact of a protein in the network on the basis of how much it is crucial for the network itself (i.e., how much its absence impairs the network). Starting from genes included in the most specific children of each significative cellular component term, we assigned a different weight to our DEGs according to their different properties within the network (Table S4). Interestingly, thanks to this analysis, DEGs included in the mitochondria had a high impact with respect to other structures (Figure 6, top frames). Moreover, considering again the fold change, mitochondria DEGs were highly inhibited in Aβ samples, whereas the CBNR pretreatment completely inverted their regulation at a level similar to those of the endoplasmic reticulum (Figure 6, bottom frames). Finally, top-weighted DEGs were genes mainly involved in oxidative phosphorylation in the mitochondria; genes involved in protein folding, glucose, and lipid metabolism in the endoplasmic reticulum; and among the intracellular complexes, some genes of the ubiquitin complex (Table 1). Therefore, the regulation of these key processes could be the way in which CBNR counteracts the Aβ damage in an AD model.

Specifically, among the top-weighted DEGs of the mitochondria, there are four upregulated genes (*COX6B1*, *OXA1L*, *MT-CO2*, and *MT-CO3*), which encode components of the cytochrome c oxidase (COX) complex. This is a heteromeric enzymatic complex, localized in the inner mitochondrial membrane, composed of three catalytic subunits encoded by mitochondrial genes (such as *MT-CO2* and *MT-CO3*) and diverse structural subunits encoded by nuclear genes (including *COX6B1* and *OXA1L*). COX, also known as Complex IV, is the last component of the mitochondrial respiratory chain, driving oxidative phosphorylation. It catalyzes the reduction of oxygen to water, cooperating with the other complexes to eject protons into the intermembrane space, creating an electrochemical gradient fundamental for ATP production by complex V (ATP synthase) [41]. Moreover, OXA1L protein is required for the insertion of integral membrane proteins into the mitochondrial inner membrane and is essential for the assembly of complexes I, IV, and V [42,43]. Defects in oxidative phosphorylation have been widely reported in AD patients and in in vivo AD models. Indeed, subunits of the COX complex are frequently inhibited in AD, leading to increased reactive oxygen species levels and reduced ATP production [44,45]. Therefore, the fact that CBNR is able to upregulate some of the main COX subunits with respect to Aβ samples supports the molecular way in which CBNR improves the viability of SH-SY5Y cells in our AD model. Regarding the endoplasmic reticulum, the *HSPA5* gene was upregulated. It encodes a member of the heat-shock protein 70 (HSP70) family, a chaperone important for the proper folding, quality control, and assembly of proteins in the endoplasmic reticulum lumen [46]. Accumulation of misfolded/unfolded proteins is a common feature of AD and is linked to apoptosis processes [7,9]. Additionally, genes related to glucose (*G6PC3*) and lipid metabolism (*HSD17B7*, *ERG28*, and *SCD*) were also upregulated by CBNR. *G6PC3* encodes the catalytic subunit of glucose-6-phosphatase (G6Pase), an enzyme localized in the endoplasmic reticulum lumen, which catalyzes the hydrolysis of glucose-6-phosphate to glucose and phosphate in the last step of glycogenolysis and gluconeogenesis. Deficiency of the enzyme is also associated with apoptosis [47]. *HSD17B7* and *ERG28* are genes involved in cholesterol biosynthesis, encoding a catalytic and non-catalytic protein, respectively [48,49,50]; in contrast, *SCD* encodes the stearoyl-CoA desaturase, an enzyme mainly involved in oleic acid biosynthesis [51]. Accumulating evidence reported lipid dyshomeostasis in AD, revealing complex and diverse mechanisms that connect lipid metabolism with AD-related pathophysiologies [52]. Notably, we also observed the downregulation of the *RYR2* gene, which encodes the ryanodine receptor 2 channel that is involved in the release of Ca^2+^ ions from the endoplasmic reticulum into the cytosol, regulating cellular calcium homeostasis. Hyperexcitability and a high concentration of cytosolic calcium are common defects in AD. Upregulation and/or overactivation of the RYR2 channel have been reported in AD models [53,54].

Finally, ubiquitin complex genes *CUL3*, *FBXW7*, and *UBE2D1* were increased among the intracellular complexes. The Cullin 3 protein encoded by the *CUL3* gene is a crucial scaffold component of the E3 ubiquitin ligase complex, which is required in the polyubiquitination and subsequent degradation of specific protein substrates [55]. The *FBXW7* gene encodes the substrate recognition component of the SCF-type E3 ubiquitin ligase, which is involved in phosphorylation-dependent ubiquitination. Remarkably, FBXW7 has been demonstrated to antagonize apoptotic JNK signaling by binding to phosphorylated JUN and promoting its ubiquitination and subsequent degradation [56]. Instead, the *UBE2D1* gene encodes a member of the E2 ubiquitin-conjugating enzyme family, which works in concert with E3 ubiquitin ligase and the E1 ubiquitin-activating complex in the ubiquitination and subsequent degradation of selected short-lived and misfolded/unfolded proteins [57]. Impairment of these enzymes is associated with AD, contributing to the accumulation of abnormal proteins [55,58].

It is important to note that we obtained diverse ribonucleoprotein genes related to ribosomes and ribosome biogenesis, which were deregulated by CBNR in our AD model. However, some of them were upregulated and others were downregulated. Moreover, there are controversial reports about the expression of translation machinery proteins in AD (details in [59,60,61]). This point will require further study.

Regarding the potential limitations of this study, our results should be further validated using in vivo models that mimic the real physiopathological conditions of AD. For instance, the impact of CBNR in vivo may be tissue-specific, and the dose effect could differ from an in vitro to an in vivo model. Accordingly, transgenic murine AD models and neurons derived from human-induced pluripotent stem cells of AD patients may be helpful.

In conclusion, our study demonstrates that the phytocannabinoid CBNR displays neuroprotective properties in an Aβ-induced AD model in differentiated SH-SY5Y cells. Indeed, it restores mitochondrial and endoplasmic reticulum dysfunctions, regulating genes related to oxidative phosphorylation, protein folding, ubiquitin-mediated degradation, and glucose and lipid metabolism. Therefore, CBNR could be a novel molecule able to prevent some of the key early features of AD and potentially other diseases characterized by similar dysfunctions.

## Figures and Tables

**Table 1:** Top 10% DEGs of each structure resulting from the Aβ vs. CBNR20-Aβ network-based transcriptomic analysis.

Structure	Gene	Fold Change	Weight	Cellular Component Term
	*COX6B1*	0.11	18.56511	GO:0005743
	*OXA1L*	0.13	13.56726	GO:0005743
	*MT-CO2*	0.17	8.746326	GO:0005743
Mitochondria	*NDUFS2*	−0.06	7.026098	GO:0098798
	*MRPS5*	−0.17	5.356617	GO:0098798
	*MT-CO3*	0.2	3.993246	GO:0005743
	*MRPL17*	0.46	3.257547	GO:0005743
	*LPIN1*	−0.07	8.420952	GO:0005789
	*PSEN1*	0.18	4.153903	GO:0005789
	*HSPA5*	0.13	3.228354	GO:0005789
Endoplasmic	*HSD17B7*	0.41	2.454414	GO:0005789
reticulum	*ERG28*	0.41	1.92561	GO:0005789
	*G6PC3*	0.13	1.795113	GO:0005789
	*RYR2*	−0.25	1.421016	GO:0005789
	*SCD*	0.08	1.242196	GO:0005789
	*DCAF13*	−0.11	12.2385	GO:0005730
	*PPP1R8*	−0.21	5.108257	GO:0016607
	*CTR9*	0.18	4.591837	GO:0016607
	*POLR2D*	0.28	3.826531	GO:0016607
	*BYSL*	−0.29	2.928742	GO:0005730
Nucleus	*TBP*	−0.14	2.919908	GO:0005730
	*NGDN*	−0.18	2.703704	GO:0005730
	*POLR1A*	−0.11	2.074889	GO:0005730
	*WDR46*	−0.19	1.969072	GO:0005730
	*MYBBP1A*	−0.16	1.881808	GO:0005730
	*POLR2E*	−0.29	10.12183	GO:0140535
	*RPS28*	−0.14	8.585967	GO:1990904
	*RPS18*	−0.21	7.982169	GO:1990904
	*BYSL*	−0.29	5.367588	GO:1990904
	*NGDN*	−0.18	4.317974	GO:1990904
	*CDKN1B*	−0.22	4.217337	GO:1990234
	*CUL3*	0.23	4.099537	GO:1990234
Intracellular	*MRPL21*	0.27	3.86925	GO:1990904
complexes	*TBP*	−0.14	3.414874	GO:0140535
	*MRPL17*	0.46	3.37295	GO:1990904
	*FBXW7*	0.14	3.17825	GO:0140535
	*TRAPPC1*	0.26	3.163546	GO.0140535
	*POLR2D*	0.28	3.12412	GO:0140535
	*RPS8*	0.08	3.123559	GO:1990904
	*RING1*	−0.33	2.57826	GO:1990234
	*UBE2D1*	0.24	2.366765	GO:1990234
	*IKBKB*	−0.20	2.145099	GO:1990234

Fold change is related to the naïve fold change obtained by the comparison of Aβ vs. CBNR20-Aβ and rounded to the second decimal place. The weight is obtained by a combination of betweenness centrality, closeness centrality, neighborhood connectivity, clustering coefficient, average shortest path, and number of vertices.

## Data Availability

The data presented in this study are openly available in the NCBI Sequence Read Archive at BioProject, accession number PRJNA1079210.

## Supplementary Materials

The following supporting information can be downloaded at: https://www.mdpi.com/article/10.3390/cells13121012/s1, Figures S1–S4: Uncropped Western blots; Table S1: Primary data of Western blot analyses and the MTT assay; Table S2: DEGs resulting from the Aβ vs. CBNR20-Aβ or CTRL vs. Aβ comparisons; Table S3: Overrepresented cellular component terms in the Aβ vs. CBNR20-Aβ comparison; Table S4: Network analysis parameters in the Aβ vs. CBNR20-Aβ comparison.
