# Supplementary figures and images for: Cannabinerol Prevents Endoplasmic Reticulum and Mitochondria Dysfunctions in an In Vitro Model of Alzheimer’s Disease: A Network-Based Transcriptomic Analysis
Source: Cells. 2024 Jun 10;13(12):1012. doi: 10.3390/cells13121012 (PMC11201759; doi:10.3390/cells13121012)

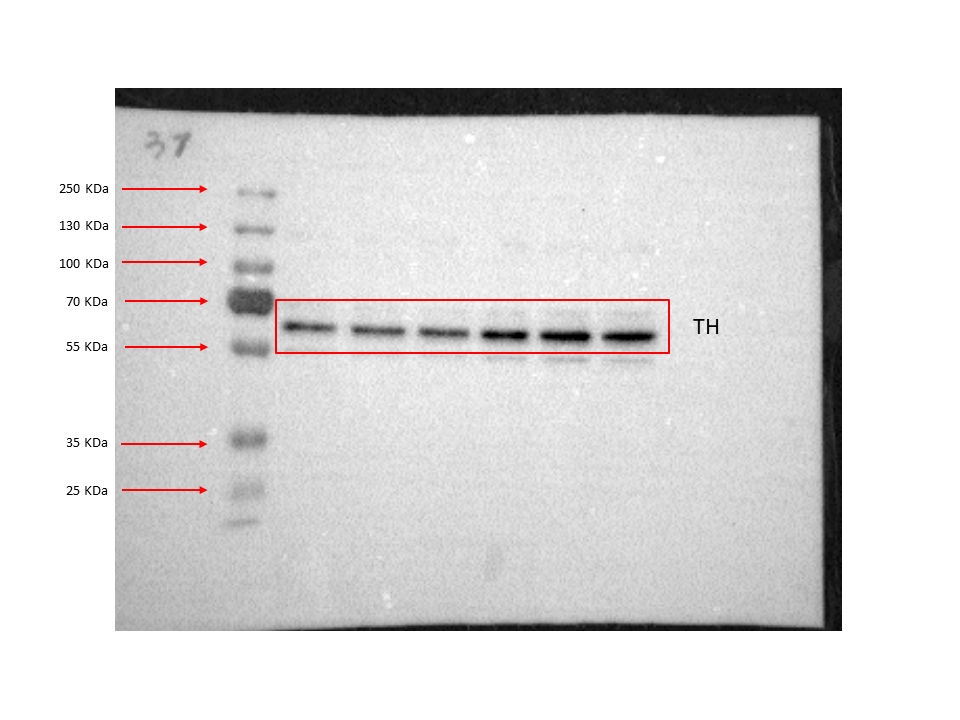

Supplement: Supplementary file 1 [file cells-13-01012-s001.zip › FigureS1.TIF]

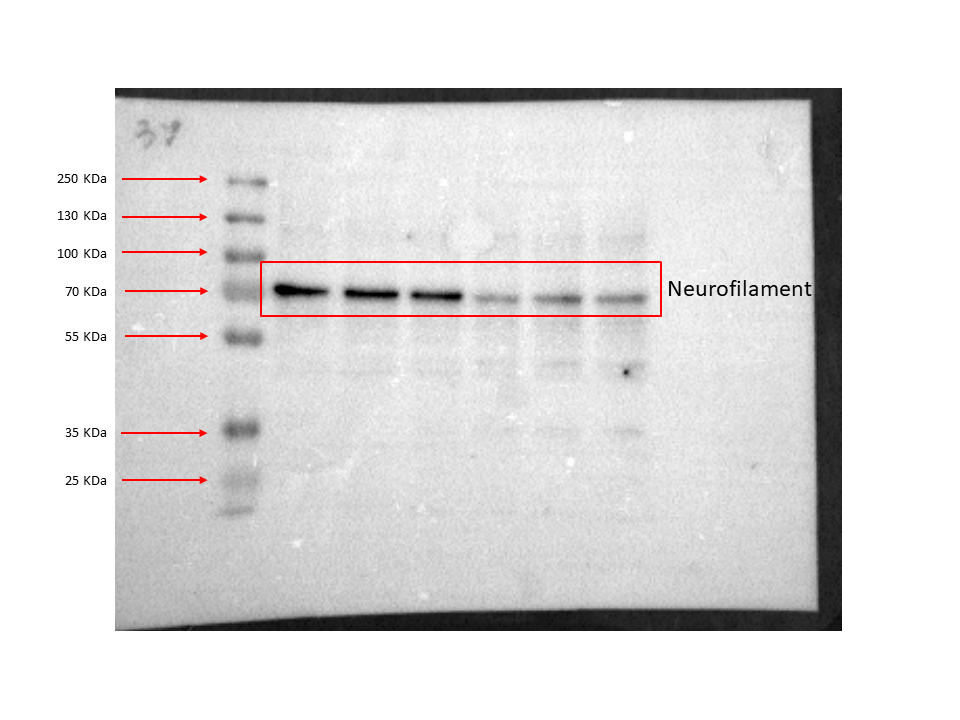

Supplement: Supplementary file 1 [file cells-13-01012-s001.zip › FigureS2.TIF]

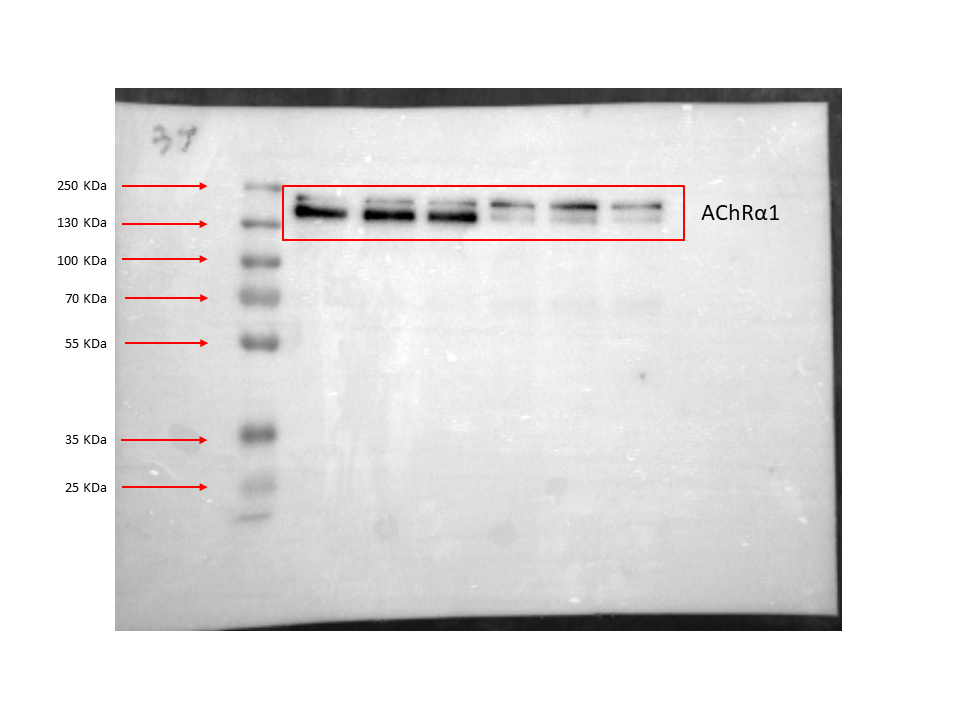

Supplement: Supplementary file 1 [file cells-13-01012-s001.zip › FigureS3.TIF]

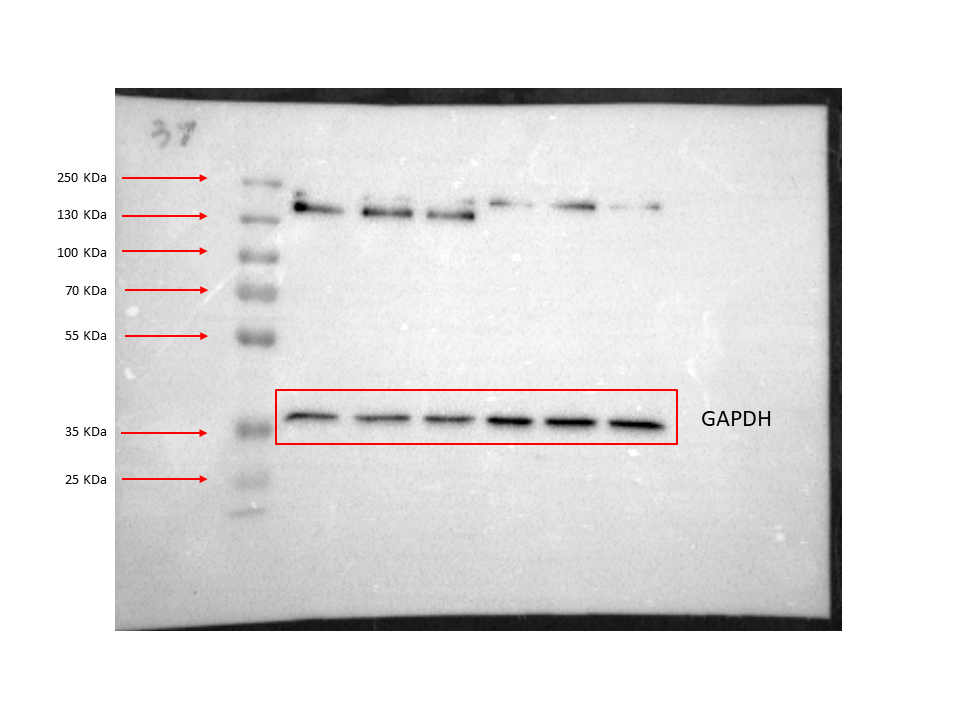

Supplement: Supplementary file 1 [file cells-13-01012-s001.zip › FigureS4.TIF]
